# Neuroimaging as a Selection Tool and Endpoint in Clinical and Pre-clinical Trials

**DOI:** 10.1007/s12975-016-0487-1

**Published:** 2016-08-20

**Authors:** Keith W. Muir, I. Mhairi Macrae

**Affiliations:** Institute of Neuroscience and Psychology, College of Medical, Veterinary and Life Sciences, University of Glasgow, Glasgow, Scotland UK

**Keywords:** (4-6 for indexing purposes) stroke, Angiography, MRI, CT, Mismatch, Rat

## Abstract

Standard imaging in acute stroke enables the exclusion of non-stroke structural CNS lesions and cerebral haemorrhage from clinical and pre-clinical ischaemic stroke trials. In this review, the potential benefit of imaging (e.g., angiography and penumbral imaging) as a translational tool for trial recruitment and the use of imaging endpoints are discussed for both clinical and pre-clinical stroke research. The addition of advanced imaging to identify a “responder” population leads to reduced sample size for any given effect size in phase 2 trials and is a potentially cost-efficient means of testing interventions. In pre-clinical studies, technical failures (failed or incomplete vessel occlusion, cerebral haemorrhage) can be excluded early and continuous multimodal imaging of the animal from stroke onset is feasible. Pre- and post-intervention repeat scans provide real time assessment of the intervention over the first 4–6 h. Negative aspects of advanced imaging in animal studies include increased time under general anaesthesia, and, as in clinical studies, a delay in starting the intervention. In clinical phase 3 trial designs, the negative aspects of advanced imaging in patient selection include higher exclusion rates, slower recruitment, overestimated effect size and longer acquisition times. Imaging may identify biological effects with smaller sample size and at earlier time points, compared to standard clinical assessments, and can be adjusted for baseline parameters. Mechanistic insights can be obtained. Pre-clinically, multimodal imaging can non-invasively generate data on a range of parameters, allowing the animal to be recovered for subsequent behavioural testing and/or the brain taken for further molecular or histological analysis.

## Introduction

Stroke is a clinical syndrome that has diverse causes, and imaging is an essential component of diagnosis. At the most basic level, brain imaging must distinguish intracerebral haemorrhage (ICH) from ischaemic stroke, since this cannot be recognised reliably on clinical grounds alone [1]. As imaging methods have evolved, information on key structural and physiological features has become widely available and many imaging techniques are now part of routine clinical care. Others are employed in clinical trials either for enhancing patient selection or as a biomarker of outcome. This review will focus on uses of imaging as a translational tool.

Brain parenchymal imaging with computed tomography (CT) or magnetic resonance imaging (MRI) forms the mainstay of clinical imaging. Radionuclide-based methods such as single-photon emission computed tomography (SPECT) or positron emission tomography (PET) offer specific physiological and molecular imaging techniques suitable for research, but radiation exposure and long acquisition and analysis times make these methods unsuited to acute clinical use.

Two main aspects of imaging are of importance in clinical studies: techniques that give an index of the ischaemic penumbra (and its related parameter, the ischaemic core) and imaging of the vasculature.

The concept of the penumbra as originally described by Astrup, Siesjo and Symon [2] related reduction in cerebral blood flow to loss of electrical activity and tissue viability and defined the concept of perfusion thresholds distinguishing reversible from irreversible loss of neuronal function. Subsequent human PET studies translated these concepts into the relationship between cerebral blood flow and the metabolic rate for oxygen [3]. These concepts can be translated to CT and MRI [4, 5]. In general, the non-viable ischaemic core on MRI corresponds to tissue with reduced apparent diffusion coefficient (ADC) of water, consequent to failure of energy-dependent membrane ion exchange and shift of interstitial water to the intracellular compartment. Reduced ADC is visualised clinically as a diffusion-weighted image (DWI). The DWI lesion on MRI corresponds closely to reduced cerebral blood flow (CBF) or cerebral blood volume (CBV) on perfusion imaging, either with MR (MRP) or with CT perfusion (CTP). The ischaemic penumbra corresponds to tissue that has less severe reduction of perfusion, and although electrically inactive (and thus causing clinical symptoms) remains viable for a period of minutes to hours if reperfused [6]. The penumbra therefore is hypoperfused, but exhibits less severe reductions in CBF, and preserved or increased CBV and normal DWI. The distinction between functionally impaired penumbra and regions of “benign oligaemia”—tissue that exhibits reduced perfusion but will survive regardless of whether or not reperfusion occurs—is generally based upon thresholds of prolonged mean transit time (MTT) or delayed time to peak (TTP) in contrast-based perfusion studies using either MRP or CTP. Definitions of these tissue compartments—core, penumbra and benign oligaemia—depend upon the imaging technique and the selection of threshold values. Most studies to define thresholds have been of small or modest size, and it is therefore unsurprising that proposed thresholds have varied across different studies and different times [7]. It is likely that the thresholds themselves vary according to the duration of ischaemia [8].

Vascular imaging with CT or MR angiography (CTA or MRA) can identify the presence of extracranial stenosis, the presence of intracranial thrombotic occlusion in large vessels (typically out to the third order branches of the middle cerebral artery [MCA]) and the extent of primary and leptomeningeal collaterals.

## Advantages and Disadvantages of Imaging in Clinical Trial Design

Inclusion of patients who lack a relevant biological target for a given intervention has a significant impact on sample size in clinical trials due to the dilution of the target responder group. As the proportion of patients with no relevant biological target increases, so the sample size increases markedly [9]. For illustration, a 25 % relative reduction in poor outcome might require a sample size of 390 per group if all patients have a biological target, but this rises to 920 per group if only 65 % have a relevant target [9]. In phase 2 clinical trials, sample size is generally small and it is particularly important to explore issues around treatment response of different groups. Imaging allows better characterisation of clinical populations than clinical criteria alone, and its use at this stage of research is recommended [10]. Clinical criteria try to exclude non-stroke diagnoses (e.g., seizures and hypoglycaemia), and basic imaging excludes some non-stroke structural central nervous system lesions. Other imaging features may be regarded as a prerequisite for patient selection in specific scenarios, such as the requirement for an arterial occlusion in a suitable target vessel in trials of endovascular mechanical thrombectomy (MT), as discussed below.

In ischaemic stroke, the penumbra is postulated to represent the therapeutic target for reperfusion or neuroprotectant therapies and penumbral imaging selection is expected to reduce sample size, by ensuring that all recruited subjects have a relevant biological target. The penalty of more stringent selection criteria is a greater exclusion rate and thus slower recruitment [9]. Based on limited imaging data available more than a decade ago, an estimated 5–7 % of clinically eligible patients were predicted to fulfil parenchymal imaging selection criteria (thus increasing the “number needed to screen” per trial recruit to 16–20, but ensuring an informative population). In the EXTEND-IA trial [11], 70/1044 (7 %) of those patients eligible for intravenous (IV) thrombolysis were selected for randomisation to additional MT or best medical care. Imaging criteria excluded a high proportion of patients, around half due to absence of relevant vessel occlusion and a quarter of those with vessel occlusion due to unfavourable perfusion imaging, suggesting that estimates for the potential impact on recruitment rates were close to reality.

Two recent phase 2 trials comparing the thrombolytic agent tenecteplase with the standard of care rtPA alteplase illustrate the advantages and disadvantages of imaging selection and endpoints. Parsons and colleagues applied imaging selection criteria including presence of CTA-defined arterial occlusion, minimum penumbra volume and maximum core volume, achieving a relatively homogeneous trial population and finding tenecteplase superior to alteplase on both clinical and imaging endpoints [12]. The ATTEST trial [13] acquired CTA and CTP but used only CT and clinical criteria to define eligibility, resulting in a more heterogeneous population. ATTEST did not demonstrate any difference in clinical or imaging outcomes. Parsons’ imaging selection criteria excluded 79 % of rtPA-eligible patients, and only 12 % of treatment-eligible patients were randomised, compared to 66 % of patients in ATTEST. In translating results from such focused phase 2 trials, phase 3 trials face the challenge of establishing generalisability in settings where complex imaging is less likely to be available, but absolute effect sizes are likely to be overestimated based on highly selected phase 2 findings.

## Angiographic Imaging

The third Interventional Management of Stroke (IMS-3) trial [14] tested the value of additional MT amongst patients treated with IV thrombolysis with recombinant tissue plasminogen activator (rtPA), but did not identify any significant benefit of MT compared to IV rtPA alone. The IMS-3 trial started when CTA was not available routinely, and consequently a majority of patients (53 %) randomised in the trial were found to have no relevant arterial occlusion when undergoing initial angiography for MT. Of those having CTA or MRA, when these became adopted into clinical practice during the course of the trial, 8 % had no arterial occlusion and a further 20 % had isolated distal vessel occlusion (beyond the size of target vessel for the interventions being undertaken). It is therefore likely that at least one third of those patients eligible on clinical criteria would have been excluded on the basis of CTA. In addition, a proportion of patients is found to have already reperfused between randomisation and the start of the endovascular procedure, (11 % in the recent EXTEND-IA trial [11]) emphasising that vessel status (and presumably also tissue status) is a dynamic phenomenon in the acute phase of stroke and that eligibility may fluctuate. A subgroup analysis of IMS-3 that included only those patients with confirmed large artery occlusion on CTA where it was available found a trend towards benefit for additional MT [15].

All subsequent (positive) trials of MT have required the presence of a relevant occlusion on non-invasive imaging, predominantly CTA. Limiting invasive procedures to those with relevant large artery occlusion is both ethically appropriate and maximises trial efficiency. The appropriate treatment target may, of course, vary according to the intervention being tested. Eligibility for MT is readily defined by widely available non-invasive investigation; for other interventions, however, this might be less readily identified and the danger is that focus on a group with a logical target excludes patients who might derive benefit through mechanisms that are not initially understood. A secondary analysis of the combined data from the EPITHET trial and observational DEFUSE study reported greater benefit of thrombolysis in the presence of a visible arterial occlusion on MRA [16]. It may therefore be that imaging-defined arterial occlusion represents a target population for reperfusion therapies in general. Since the majority of major trials of intravenous thrombolysis were undertaken without vascular imaging, it is not possible to define whether the benefit of IV thrombolysis differs amongst those without visible occlusion.

## Perfusion Imaging

Observational data suggest that reperfusion confers clear benefit amongst those with variously defined penumbral imaging patterns on either MRI or CT, whereas reperfusion does not have a clear benefit amongst those without such a pattern [17–19]. In some studies, the time window for potential benefit from reperfusion appeared longer than that based from conventional imaging alone, supporting study designs that are investigating the potential efficacy of reperfusion in imaging-selected patients at later time points [20]. The number of subjects included in these observational studies, however, is small; in DEFUSE-2, only 21 patients contributed to the “no target mismatch” group [17]. These patients were already highly selected by being considered eligible for endovascular MT and also being MRI-compatible, and generalisability of these observations is accordingly unclear.

In part, the differences in outcome in observational studies may be explainable by the reduced risk of symptomatic intracerebral haemorrhage (SICH) when those patients with “large core” profiles on imaging are excluded. Large ischaemic core lesions (defined by DWI lesion, reduced CBF or CBV, or hypoattenuation on CT) generally exceed 50 or 70 ml in volume. The Alberta Stroke Programme Early CT [ASPECT] score offers an alternative for non-contrast CT. An ASPECT score <7 was used to exclude large core patients in several recent endovascular trials. Possible increased risk of SICH when reperfusion occurs in patients with MRI-defined “large core” has been reported in the DEFUSE and EPITHET datasets [21]. CTP-defined large core is associated with higher incidence of ICH regardless of reperfusion [22]. Reduced incidence of SICH has been reported in observational cohorts with both MRI [23] and with perfusion CT [19], but definitions of core have been variable [7, 24, 25] and standard criteria are not yet agreed.

Several trials propose that the “penumbral” or “target mismatch” pattern on imaging defines the presence of viable tissue in a largely time-independent manner, viable tissue persisting for longer periods than the conventional treatment window for IV thrombolysis for example. Trial designs have therefore sought to select patients with penumbral imaging patterns at late time points (e.g., 3–9 h after symptom onset in the DIAS and DEDAS trials [26, 27], 4.5–9 h in the ongoing ECASS-4 and EXTEND trials). Issues with consistency of imaging processing and interpretation across multiple sites with different approaches to scanning have confounded these studies [7, 20–30].

Penumbral imaging parameters have been used in three recent MT trials to select patients as a means of minimising the sample size by excluding those with very favourable or very poor prognosis [11, 12, 26–28, 30, 31], a concept proposed originally from PET observations [3]. The SWIFT-Prime [31] and EXTEND-IA [11] trials used perfusion imaging (predominantly CTP) and ESCAPE [32] using multiphase collateral vessel imaging. These three trials found larger effect sizes compared to the two MT trials that used only CT and CTA for selection, MR CLEAN [33] and REVASCAT [34] (Table 1).

**Table 1:**
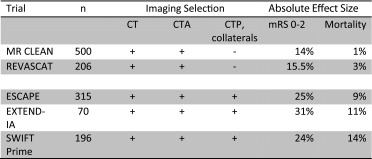
Effect sizes in published endovascular thrombectomy trials

Reliable evidence that parenchymal imaging parameters modify the treatment effect of a given intervention is, however, lacking and indeed, in the few trials that have acquired complex imaging but not used imaging selection parameters, there was no evidence of a differential treatment effect [33, 35–38]. This was evident most recently from the CTP subgroup analysis of the MR CLEAN trial, where CTP was acquired in 175/500 (35 %) patients but not used explicitly to select patients. Secondary analysis identified no heterogeneity of the treatment effect of MT with any perfusion parameter [38]. It is possible that this may reflect a dominant effect of very early reperfusion and that selection by penumbral imaging might be of greater value in later time windows.

The MR CLEAN sub-study emphasised a further concern that despite acquisition in most patients (67 % in all), data could not be analysed due to technical limitations in more than one third of cases. Since technical factors such as motion artefacts and premature truncation of the venous outflow curve are more likely in those with greater stroke severity, there may be additional selection bias introduced. Disagreement between local observers’ interpretation of perfusion scans and central “expert” interpretation is also common, even in highly experienced centres [19]: in an observational cohort study using CTP to select patients for IV thrombolysis, 25 % of patients were inappropriately excluded from IV rtPA on the basis of discrepant CTP interpretation and conversely 27 % who should have been excluded were treated with IV rtPA. The lack of standardisation for perfusion imaging has been well documented [7, 25] and is considered to have contributed to the failure of at least two clinical trials [28–30].

## Imaging as a Selection Tool in the Absence of Clinical Information

Imaging may provide information about onset time when clinical history is unable to, for example the 25 % of patients who wake with symptoms, or those unable to give a history because of dysphasia or conscious level reduction and no witness.

The ongoing WAKE-UP trial [39] uses the difference in signal intensity between diffusion-weighted MRI (immediate signal change due to cytotoxic oedema) and T2 FLAIR (slower and gradual change as vasogenic oedema develops) to identify likely onset of ischaemia in the preceding 4.5 h and selects patients with this pattern to be randomised to IV rtPA or placebo [40]. Similar proposals using non-contrast CT have been advanced [40, 41]. Time-independent imaging parameters, such as a presence of a penumbral pattern on CTP, are utilised in the current ECASS-4 and EXTEND trials [42], which enrol stroke of unknown onset time (in addition to those clearly presenting in the 4.5–9-h time window).

## Imaging as an Explanatory Factor rather than Selection Filter

Imaging features such as large ischaemic core, poor leptomeningeal collateral vessels, volume of hypoattenuation on non-contrast CT, volume of tissue with very severe hypoperfusion [43] or perfusion lesion volume [44] are strongly predictive of outcome and independent of clinical features. Characterising the site of arterial occlusion, penumbra and core volumes in the phase 2 Alteplase-Tenecteplase Trial for Efficacy in Stroke Thrombolysis (ATTEST) study offered insights into chance imbalances between treatment groups that were not apparent from the clinical assessments alone [13]. Acquisition of advanced penumbral and vascular imaging also allows pooling of data with other phase 2 studies that share similar imaging protocols. It is therefore a recommendation of the STAIR Imaging Roadmap meeting that phase 2 trials in acute stroke should wherever feasible gather such data. Trials where imaging has been acquired but not used to select patients, such as in ATTEST, may offer a more generalizable treatment effect estimate to take forward to phase 3 trials, as well as suggesting selection criteria that might be utilised to maximise trial efficiency. More highly selected patient groups, such as studied in the Australian phase 2 tenecteplase trial [12], can achieve highly encouraging results from small sample sizes, but inevitably overestimate the absolute effect size that would be seen in a less selected population.

## Imaging as an Endpoint in Clinical Trials

Whilst day 90 or later time points are generally used for clinical outcome assessment, efficacy measures that can provide earlier information are valuable in many clinical trial designs. Imaging biomarkers may be particularly valuable in dose-finding studies with an adaptive design, where ineffective or unsafe doses can be discarded during the conduct of the trial as data accrue. An early example of this approach was a phase 2/3 trial of tenecteplase that compared three different tenecteplase doses against a standard alteplase (rtPA) dose [45], using early clinical improvement and 24-h CT safety outcome (incidence of SICH) to guide dose selection dynamically. Alternative imaging metrics have been proposed, including “final” infarct volume on MRI [46], reperfusion [12] or early recanalization.

The incidence of ICH on follow-up imaging has been a key safety parameter in trials of thrombolysis or reperfusion. Brain oedema is another safety parameter that has been reported only infrequently.

There are significant difficulties with several proposed imaging biomarkers. Selection bias, as noted above, is an issue. Patients with exclusions based on safety in MRI or in relation to contrast agent administration, those who do not tolerate imaging, or who die before follow-up imaging are all excluded from recruitment, analysis or both. There is a significant inter-observer variability in measurement of volume parameters, worse for CT than for MRI [47], and the occurrence of brain swelling, intracerebral haemorrhage and presence of established background ischaemic changes may both obscure any volumetric assessment and vary according to the time of follow-up imaging acquisition. Very late “outcome” imaging is confounded by tissue atrophy.

A summary of the main points for and against the use of MRI for stroke patient selection and stroke endpoints is displayed in Table 2 on previous page.

**Table 2:**
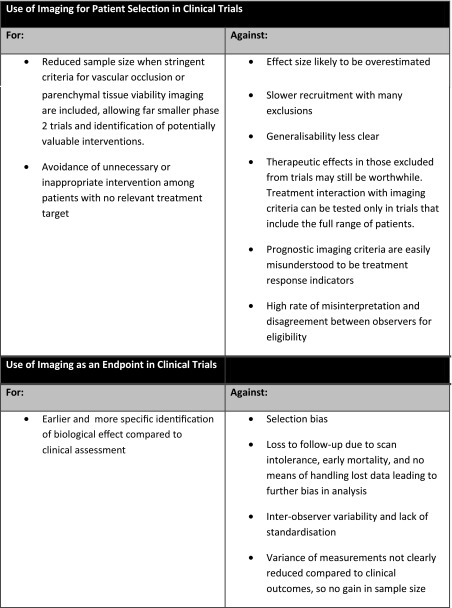
Clinical summary

## Pre-clinical

Scaled down equipment exists for small animal imaging using PET, MRI and functional ultrasound. MRI is currently the most commonly used technique offering non-invasive multimodal imaging with good resolution and without the need for ionising radiation. The range of sequences available and the ability to scan the animal on a number of occasions and repeat scans over a number of hours within a single scanning session have provided pre-clinical stroke research with a very powerful tool. It has been used to study stroke pathophysiology, to develop more informative diagnostic imaging and to investigate new therapies targeted at acute and chronic phases of stroke. MRI imaging available to the basic scientist includes DWI for ischaemic injury and peri-infarct depolarisations, perfusion imaging (PI) for perfusion deficits, and combination of the two for identification of tissue-at-risk, MR spectroscopy for metabolites of interest, tissue pH and sodium, T2* with contrast for blood–brain barrier breakdown, T2 for final infarct, FLAIR for cerebral haemorrhage, manganese-enhanced MRI for tagging and tracking injected cells and CNS connectivity studies, diffusion tensor imaging (DTI) for studying white matter injury and Fluorine 19 MR spectroscopy for imaging inflammation. Surrogate markers for recovery after stroke include DTI and T2* for functional MRI (fMRI) and baseline connectivity studies.

### Acute Stroke Imaging—Diffusion-weighted Imaging

The most informative scanning technique in the acute stroke period must surely be diffusion-weighted imaging (DWI). Using rodent middle cerebral artery occlusion (MCAO) models, focal ischaemia can be induced within the scanner and hyperintensity appears within 10 min of onset, reflecting a similar timescale to human stroke [48]. One topic of debate within the literature has been whether the DWI lesion, referred to as the “ischaemic core,” reflects irreversibly damaged tissue destined to infarct, or injured tissue, some of which is capable of recovery when promptly reperfused [49]. Early hyperintensity, reflecting restricted diffusion of water in the extracellular compartment, is partially or completely reversed by prompt restoration of flow through the MCA [50]. Reperfusion-induced reversal of some of the hyperintense lesion on DWI scans occurs in rodent stroke models (e.g., 30–90-min intraluminal filament MCAO) with recovered tissue showing a normal MR signature on the subsequent T2 images 7 days post-stroke [51, McCabe C, personal communication]. The longer the tissue is exposed to ischaemia, the greater the certainty that the DWI lesion represents irreversibly damaged tissue. With maintained MCAO, the intensity and size of the DWI lesion increases over the first 4–6 h [52] as penumbral tissue succumbs to the lack of oxygen and nutrients and the waves of spreading depolarisation that exhaust its remaining ATP stores [53, 54]. Without intervention, presumed penumbral tissue in rodent models (defined from PWI/DWI mismatch) has a lifespan of around 4–6 h [55]. However, in studies where anaesthetised, ventilated animals are monitored and blood pressure and blood gases maintained within normal limits; scanning over the first 6–7 h after MCAO in some studies has revealed minimal DWI lesion growth and prolonged penumbral lifespan [56, 57].

In pre-clinical stroke research, DWI scans are routinely processed to produce apparent diffusion coefficient (ADC) maps. This allows the impedance of water molecule diffusion in normal and injured tissue to be assigned a quantitative value (mm^2^/s) and ADC thresholds applied to provide unbiased assessment of tissue-at-risk of infarction [58]. These ADC thresholds are derived for specific time points after a maintained ischaemic insult (permanent MCAO) using final infarct volume data on later T2 scans or histology sections. There is a good agreement amongst different pre-clinical groups that has established in-house ADC thresholds (see Table 1 in reference [55]).

When studying new therapeutic interventions, an acute DWI scan, carried out after stroke onset but before delivering the therapy or reperfusion, is extremely valuable. It confirms a successful ischaemic insult in each animal and provides baseline data of ischaemic damage to assess how an intervention influences the subsequent evolution of ischaemic damage in each animal. Given the variability in lesion size observed in rodent models of MCAO, particularly with the intraluminal filament model, this allows the effect of treatment/reperfusion to be more accurately assessed by determining change in lesion size between the initial DWI lesion and final infarct, within each animal.

### Acute Stroke Imaging–Angiography and Perfusion Imaging

In contrast to clinical imaging, contrast-based angiography and perfusion sequences are rarely used in pre-clinical stroke research. Instead, non-invasive, non-contrast-enhanced approaches are used where flowing blood replaces exogenous contrast agents (e.g., time-of-flight angiography and continuous and pseudo-continuous arterial spin labelling-ASL). Angiography sequences reveal completeness of MCAO and quality of reperfusion on subsequent recanalisation of the MCA in transient MCAO models. This allows failed or incomplete MCAO to be identified early providing another way in which variability in drug studies can be controlled.

ASL techniques can provide fully quantitative CBF data (mL/100 g/min) rather than the delay-based parameters favoured clinically in the setting of dynamic susceptibility contrast imaging using gadolinium contrast. By employing an endogenous contrast agent, serial and repeat assessment of CBF is possible plus the absence of exogenous contrast agents means there is no influence on other sequences within a multimodal scanning protocol. However, ASL sequences require much longer scanning times than DWI so it is not always possible to acquire sufficient ASL slices for full coverage of the MCA territory during serial (e.g., hourly) multimodal imaging. Few studies have confirmed the accuracy of the technique on individual scanners [59, 60], and values recorded for non-ischaemic (contralateral) CBF following MCAO in rats show wide variations in CBF values (see Table 1 in reference [55]).

General anaesthesia (GA) represents an additional complication and source of variation in CBF values in rodent stroke studies. GA will influence blood pressure and in unventilated animals, respiration and blood gases. These in turn can influence CBF, particularly in experimental stroke studies where autoregulation will be impaired. Therefore, in contrast to the consistency in defining ADC thresholds and the ischaemic lesion from DWI scans, there is greater uncertainty in defining CBF thresholds for assessment of the perfusion deficit from ASL scans. Problems can be encountered in defining in-house perfusion thresholds, and published perfusion thresholds range from 57 to 81 % reduction in flow (see Table 1 in reference [55]). For example, following permanent MCAO, visual inspection of colour-coded CBF maps at 4 h post-stroke alongside final T2 infarct scans at 24 h can show regions of severely hypoperfused perfusion deficits which are larger than the infarct on the same slice, making it problematic to define perfusion thresholds based on final, oedema-corrected infarct. It is also likely that CBF in the ischaemic hemisphere will rise on removal of GA, with collateral flow improving as blood pressure rises, potentially resetting the boundaries between normal flow, benign oligaemia, penumbra and ischaemic core.

Serial perfusion imaging (PI) following both permanent and transient MCAO allows for the assessment of temporal changes in perfusion following intervention. By combining PI and ADC maps, the amount of PI/DWI mismatch is used to assess the lifespan of this “tissue-at-risk” and the influence of interventions. It also facilitates the selection of animals to balance groups prior to randomization and entry into subsequent therapy studies.

### Acute Stroke Imaging—Determining Tissue Viability and Stroke Onset Time

Stroke onset time, key in the evaluation and management of acute stroke patients, is often unavailable. Around 25 % of patients fall within the “wake up” category [61] excluding them from access to acute reperfusion therapy. Therefore, pre-clinical research has explored the possibility of imaging biomarkers which could provide a reasonable estimate of stroke onset time. One proposed biomarker is brain tissue sodium concentration (TSC) which appears to increase linearly over time in tissue at risk of infarction with a threshold for tissue viability close to normal values. Since brain tissue sodium can be measured with MRI in man, a single acute stroke TSC scan could theoretically be used to extrapolate back to estimate stroke onset time which would coincide with the time point at which tissue sodium signal starts to rise above normal [62, 63]. However, subsequent serial animal MRI studies testing this hypothesis found extrapolating ischaemic core data back in time resulted in differences of >1 hour between actual stroke onset time and the onset of elevated TSC [57]. Nonetheless, TSC may serve as a useful biomarker of tissue viability since differing TSC and temporal profiles are present in ischaemic core (linearly increasing TSC) and penumbra (≤ contralateral TSC) [57]. This could be a useful adjunct to PI/DWI mismatch, commonly used pre-clinically to identify potentially savageable penumbral tissue but still lacking consensus on the setting of perfusion and diffusion thresholds which are also likely to vary according to time from stroke onset. More direct assessment of tissue viability following stroke has been proposed by exploiting the T2* BOLD sequence and the different magnetic properties of oxy- and deoxyhaemoglobin. When combined with an oxygen challenge, the T2* signal change (as paramagnetic deoxyhaemoglobin is converted to diamagnetic oxyhaemoglobin) is greater in penumbra than in adjacent ischaemic core and normal tissue [64–66]. Since the basis for increased signal in penumbra is due to an increased oxygen extraction factor, vascular changes associated with the change in PaO_2_, or a combination of the two, this could represent a more valid method for detection of penumbra.

A summary of the main points for and against the use of MRI for stroke animal selection and stroke endpoints is displayed in Table 3 on the previous page.

**Table 3:**
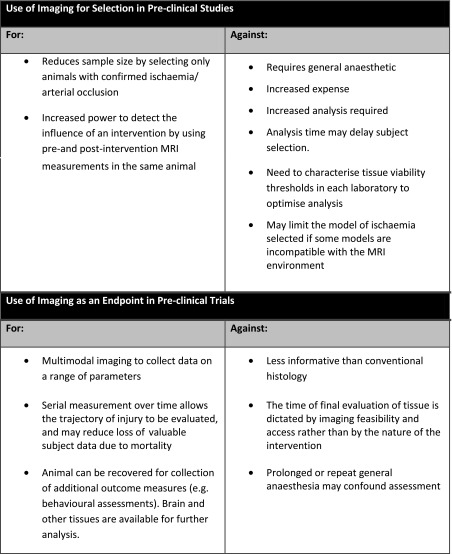
Pre-clinical summary
